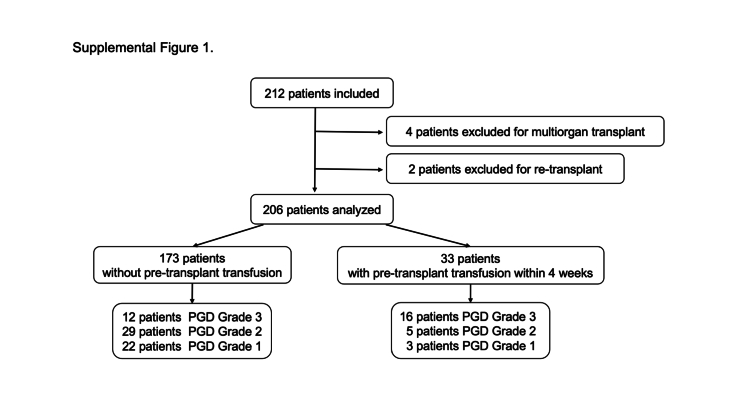# The Risk of Pretransplant Blood Transfusion for Primary Graft Dysfunction After Lung Transplant

**DOI:** 10.1016/j.atssr.2024.02.004

**Published:** 2024-03-05

**Authors:** Taisuke Kaihou, Takahide Toyoda, Emily Cerier, Yuriko Yagi, Adwaiy Manerikar, Benjamin Louis Thomae, Viswajit Kandula, Ankit Bharat, Chitaru Kurihara

**Affiliations:** 1 Division of Thoracic Surgery, Department of Surgery, Northwestern University Feinberg School of Medicine, Chicago, Illinois

## Abstract

**Background:**

Primary graft dysfunction (PGD) is the leading cause of short- and long-term mortality associated with lung transplantation. The impact of pretransplantation blood transfusions for recipients is not fully elucidated.

**Methods:**

This is a retrospective review of 206 consecutive lung transplantations performed at a single academic center (Northwestern University Feinberg School of Medicine, Chicago, IL) from January 2018 to July 2022. Data on patient characteristics, pretransplantation laboratory values, transfusion requirements, and intraoperative and postoperative outcomes were collected.

**Results:**

PGD grade 3 (PGD 3) occurred in 13.2% of the cohort (n = 28). A total of 33 patients received a blood transfusion within 4 weeks, whereas 21 patients received a blood transfusion a week before their lung transplant. Pretransplantation transfusions were strongly associated with a higher incidence of PGD 3 (48.5% vs 6.9%; *P* < .001). There was no significant difference in 1-year survival between the pretransplantation transfused group and the nontransfused group (77.7% vs 88.0%; *P* = .478). The 1year survival was reduced in recipients with PGD 3 compared with recipients without PGD 3 (63.5% vs 89.9%; *P* = .0012). In univariate analysis, pretransplant and intratransplant predictors of PGD 3 included younger age (*P* < .01), pretransplant extracorporeal membrane oxygenation (ECMO) use (*P* < .001), higher lung allocation score (*P* < .001), coronavirus disease 2019 (COVID-19)–related acute respiratory distress syndrome (*P* < .01), blood transfusion within 4 weeks (*P* < .001), longer operative time (*P* < .001), intratransplant blood transfusion (*P* < .001), and intratransplant venoarterial ECMO use (*P* < .001).

**Conclusions:**

Pretransplantation blood transfusions could be associated with a higher rate of PGD. The findings indicated the potential risks of pretransplantation blood transfusions in lung transplant recipients.

**In Short**

▪ Pretransplant blood transfusions could be attributed to a higher rate of primary graft dysfunction after lung transplantation.
▪ Clinicians should carefully evaluate the necessity of transfusions and explore alternative strategies to minimize the risk of primary graft dysfunction.

Lung transplantation is a lifesaving procedure for individuals with end-stage lung diseases. Nevertheless, despite these improvements, lung transplant outcomes remain inferior to those of other solid organ transplants.^1^ One of the most important drivers of these worse outcomes is primary graft dysfunction (PGD).^2^ PGD contributes to heightened morbidity, mortality, and graft loss, necessitating a deeper understanding of its cause and associated risk factors.

An intraoperative blood transfusion has emerged as a notable risk factor for PGD. Investigators reported a significant association between an intraoperative blood transfusion and an increased risk of PGD. Additionally, lung transplant recipients who received donor lungs with blood transfusions had an increased risk of PGD compared with recipients who did not receive such transfusions.^4^ However, the impact of pretransplantation blood transfusions for recipients is not fully studied yet.^3^^,^^5^^,^^6^ This study aimed to explore the association between pretransplantation blood transfusions and the risk of PGD.

## Patients and Methods

### Study Design

This study was approved by the Northwestern University Institutional Review Board (IRB) (STU00213616), and the IRB approved waiver of consent. This is a retrospective review of 206 patients who underwent lung transplantation at a single, high-volume, academic center (Northwestern University Feinberg School of Medicine, Chicago, IL) from January 2018 to July 2022.

### Statistical Analysis

Recipient and donor demographics, and intraoperative and postoperative outcomes, were compared between those patients with and without preoperative blood transfusions within 4 weeks. *P* values <.05 were accepted as statistically significant. EZR software (Saitama Medical Center, Jichi Medical University), a graphic user interface for R software (The R Foundation for Statistical Computing), was used to perform all analyses.

## Results

### Patient Demographics and Outcomes

A total of 212 patients underwent lung transplantation during this period in our institution. Of these patients, 206 met inclusion criteria, and 33 (16.0%) had preoperative blood transfusions within 4 weeks (Supplemental Figure). Pre–lung transplantation characteristics of the study cohort are shown in Table 1. Preoperative blood transfusion groups had a significantly higher percentage of pretransplantation use of extracorporeal membrane oxygenation (ECMO) (1.7% vs 87.9%; *P* < .001; Supplemental Text), bilateral lung transplantation (56.6% vs 93.9%; *P* < .001), and lung allocation scores (51.3 ± 16.1 vs 83.7 ± 13.2; *P* < .001) in recipient factors.

**Table 1:** Characteristics of Lung Transplant Recipients and Donors With and Without Preoperative Blood Transfusion

Variable	No Transfusion Within 4 Weeks (n = 173)	Transfusion Within 4 Weeks (n = 33)	*P* Value
Recipient factors			
Age, y	59.2 ± 11.3	48.2 ± 13	<.001
Female	78 (45.1)	14 (42.4)	.93
BMI, kg/m^2^	25.9 ± 4.7	25.2 ± 4.2	.45
BSA, m^2^	1.9 ± 0.2	1.8 ± 0.2	.66
Smoking history	77 (44.5)	9 (27.3)	.10
Hypertension	87 (50.3)	14 (42.4)	.52
Diabetes	62 (35.8)	9 (27.3)	.45
Chronic kidney disease	15 (8.7)	2 (6.1)	.88
Pretransplant ECMO use	3 (1.7)	29 (87.9)	<.001
Pretransplant plasmapheresis	13 (7.5)	2 (6.1)	1.00
Bilateral	98 (56.6)	31 (93.9)	<.001
LAS	51.3 ± 16.1	83.7 ± 13.2	<.001
Etiology			
COPD	39 (22.5)	1 (3)	.02
ILD	76 (43.9)	4 (12.1)	<.01
ARDS (COVID-19)	16 (9.2)	25 (75.8)	<.001
PAH	26 (15)	0 (0)	.04
Others	16 (9.2)	3 (9.1)	1.00
Laboratory			
Hemoglobin, g/dL	11.7 ± 2.3	8.1 ± 1.9	<.001
WBC, 1000/mm^3^	9.7 ± 3.8	11 ± 4.3	.10
PLT, 1000/mm^3^	251.8 ± 95.1	191.5 ± 129.3	<.01
Sodium, mEq/L	139.7 ± 3	141.3 ± 3.8	<.01
BUN, mg/dL	15.7 ± 6.4	22.4 ± 13.6	<.001
Creatinine, mg/dL	0.77 ± 0.22	0.61 ± 0.25	<.001
AST, U/L	27.3 ± 20.1	35 ± 31.2	.09
ALT, U/L	19.9 ± 18.7	21.8 ± 16	.63
Albumin, g/dL	3.9 ± 0.5	3.5 ± 0.7	<.001
Total bilirubin, mg/dL	0.6 ± 0.4	1.1 ± 1.2	<.001
INR	1.1 ± 0.2	1.2 ± 0.2	.03
PRA	75 (43.4)	17 (51.5)	.50
DSA	21 (12.1)	6 (18.2)	.40
Arterial blood gas			
pH	7.37 ± 0.07	7.41 ± 0.07	<.001
Paco_2_, mm Hg	50.4 ± 11.9	48.7 ± 13.1	.48
Pao_2_, mm Hg	288.5 ± 115.4	173 ± 103.9	<.001
Donor factors			
Age, y	32.8 ± 11.9	33.8 ± 12.8	.68
Female	57 (32.9)	11 (33.3)	1.00
Cause of death			
Anoxia	71 (41)	10 (30.3)	.34
Head trauma	65 (37.6)	15 (45.5)	.51
Other	37 (21.4)	8 (24.2)	.89

Values are mean ± SD or n (%).

ALT, alanine transaminase; ARDS, acute respiratory distress syndrome; AST, aspartate transaminase; BMI, body mass index; BSA, body surface area; BUN, blood urea nitrogen; COPD, chronic obstructive pulmonary disease; COVID-19, coronavirus disease 2019; DSA, donor-specific antibody; ECMO, extracorporeal membrane oxygenation; ILD, interstitial lung disease; INR, international normalized ratio; LAS, lung allocation score; PAH, pulmonary artery hypertension; PLT, platelet; PRA, panel reactive antibody; WBC, white blood cell.

### Intraoperative and Postoperative Outcomes Associated With Preoperative Blood Transfusion

Intraoperatively, patients with preoperative blood transfusions had significantly higher operative times (6.6 ± 1.9 hours vs 9.1 ± 1.6 hours; *P* < .001), ischemic times (4.9 ± 1.7 hours vs 5.7 ± 1.1 hours; *P* < .01), venoarterial ECMO use (56.6% vs 87.9%; *P* < .01), and intraoperative blood transfusions of packed red blood cells (1.4 ± 2.3 U vs 11.1 ± 7.1 U; *P* < .001), fresh frozen plasma (0.4 ± 1.1 U vs 5.1 ± 5.8 U; *P* < .001), and platelets (0.2 ± 0.7 U vs 2.9 ± 2.7 U; *P* < .001) than patients without preoperative blood transfusions (Table 2).

**Table 2:** Intraoperative and Postoperative Outcomes of Lung Transplant Recipients With and Without Preoperative Blood Transfusion

Variable	No Transfusion Within 4 Weeks (n = 173)	Transfusion Within 4 Weeks (n = 33)	*P* Value
Intraoperative outcome			
Operative time, h	6.6 ± 1.9	9.1 ± 1.6	<.001
Intraoperative blood transfusion, pRBC	1.4 ± 2.3	11.1 ± 7.1	<.001
Intraoperative blood transfusion, FFP	0.4 ± 1.1	5.1 ± 5.8	<.001
Intraoperative blood transfusion, PLT	0.2 ± 0.7	2.9 ± 2.7	<.001
Ischemic time, h	4.9 ± 1.7	5.7 ± 1.1	<.01
VA ECMO use	98 (56.6)	29 (87.9)	<.01
VA ECMO time, h	3.1 ± 1.3	3.6 ± 1.2	.08
Postoperative outcome			
Postoperative ECMO use	12 (6.9)	23 (69.7)	<.001
Reoperation	23 (13.3)	16 (48.5)	<.001
PGD 3	12 (6.9)	16 (48.5)	<.001
AKI	60 (34.7)	22 (66.7)	<.01
Dialysis	17 (9.8)	12 (36.4)	<.001
Stroke	5 (2.9)	1 (3)	1.00
Bowel ischemia	1 (0.6)	1 (3)	.30
Digital ischemia	4 (2.3)	2 (6.1)	.25
Posttransplant ventilator, d	2 (1-3)	9 (1-17)	<.001
ICU stay, d	8 (5-14)	20 (13-28)	<.001
Chest tube drainage, d	12 (9-19)	19 (14-29)	<.001
Hospital stays, d	16 (11-24)	35 (22-39)	<.001

Values are mean ± SD, n (%), or median (interquartile range).

AKI, acute kidney injury; ECMO, extracorporeal membrane oxygenation; FFP, fresh frozen plasma; ICU, intensive care unit; PGD 3, primary graft dysfunction grade 3; PLT, platelet; pRBC, packed red blood cell; VA, venoarterial.

Postoperatively, patients with preoperative blood transfusions were significantly more likely to have PGD grade 3 development (48.5%), acute kidney injury, and dialysis (66.7% and 36.4%, respectively) compared with patients without preoperative blood transfusions (6.9%, 34.7%, and 9.8%; *P* < .001, *P* <0.01, and *P* <.001, respectively) (Table 2; Supplemental Text).

There were no significant differences in posttransplant survival between those patients with or without preoperative blood transfusions within 4 weeks (1-year survival: 77.7% vs 88.0%; *P* = .478) (Figure A). PGD grade 3 occurred in 28 (13.2%) patients. The 1-year survival was reduced in recipients with PGD grade 3 compared with recipients without PGD grade 3 (1-year survival: 63.5% vs 89.9%; *P* = .0012) (Figure B).

**Figure:**
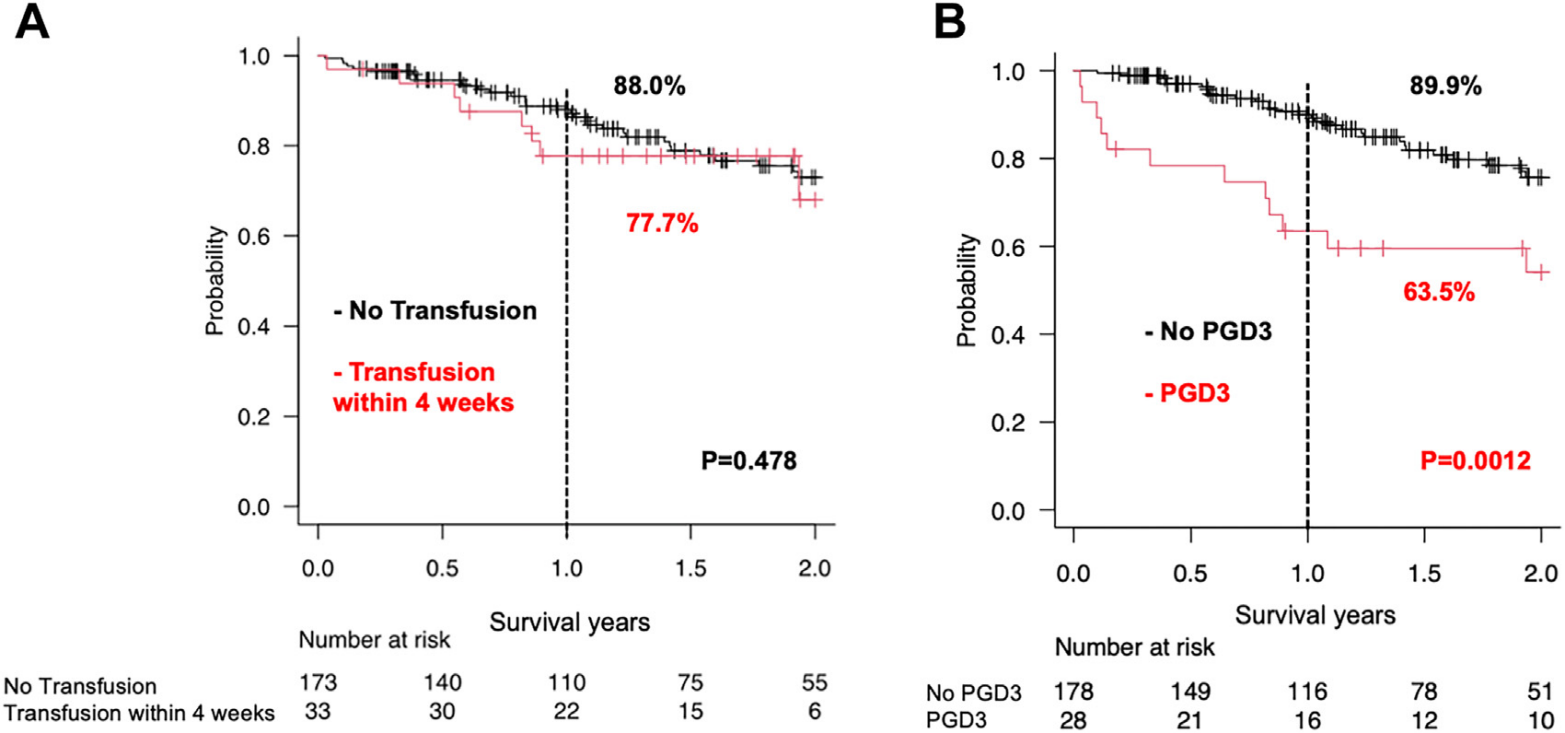
Kaplan-Meier analysis of overall survival after lung transplantation. (A) Comparison of the survival rates between recipients with a blood transfusion within 4 weeks and recipients with no transfusion. (B) Comparison of the survival rates between recipients with primary graft dysfunction grade 3 (PGD 3) and no PGD 3.

### Predictors of Primary Graft Dysfunction Grade 3

Univariate logistic regression analysis of recipient, donor, and surgical factors revealed that pretransplantation blood transfusion within 4 weeks (odds ratio [OR], 12.6; 95% CI, 5.13-31.1; *P* < .001) was a predictor of PGD grade 3 development (Table 3). The analysis also demonstrated that younger age (OR, 0.96; 95% CI, 0.93-0.99; *P* < .01), pretransplant ECMO use (OR, 12.7; 95% CI, 5.06-31.6; *P* < 001), higher lung allocation scores (OR, 1.04; 95% CI, 1.02-1.06; *P* < .001), recipient cause of acute respiratory distress syndrome (coronavirus disease 2019 [COVID-19]) (OR, 3.19; 95% CI, 1.36-7.50; *P* < .01), operative time (OR, 1.58; 95% CI, 1.23-2.02; *P* < .001), and intraoperative blood transfusions of packed red blood cells (OR, 1.18; 95% CI, 1.10-1.27; *P* < .001), fresh frozen plasma (OR, 1.26; 95% CI, 1.12-1.42; *P* < .001), and platelets (OR, 1.71; 95% CI, 1.32-2.21; *P* < .001) were predictive of PGD grade 3 development. In the multivariate analysis, preoperative blood transfusion was not an independent risk factor (OR, 6.29; 95% CI, 0.40-99.5; *P* = .19) (Table 3).

**Table 3:** Univariate and Multivariate Logistic Regression Analysis of Recipients, Donor, and Surgical Factors as Predictors of Primary Graft Dysfunction Grade 3

Variable	Univariate			Multivariate		
	OR	95% CI	*P* Value	OR	95% CI	*P* Value
Recipient factors						
Age	0.96	0.93-0.99	<.01	0.99	0.95-1.03	.65
Female	2.12	0.94-4.79	.07	…	…	…
BMI, kg/m^2^	1.00	0.92-1.09	.99	…	…	…
BSA, m^2^	0.47	0.08-2.81	.41	…	…	…
Smoking history	0.75	0.33-1.71	.49	…	…	…
Hypertension	0.89	0.40-1.97	.77	…	…	…
Diabetes	1.07	0.46-2.45	.88	…	…	…
Pretransplant ECMO use	12.7	5.06-31.6	<.001	2.22	0.13-36.9	.58
Pretransplant plasmapheresis	2.53	0.75-8.59	.14	…	…	…
Bilateral	1.94	0.79-4.81	.15	…	…	…
LAS	1.04	1.02-1.06	<.001	1.01	0.97-1.05	.68
Pretransplant blood transfusions within 4 wk	12.6	5.13-31.1	<.001	6.76	0.62-74.0	.12
Etiology						
ILD	1.02	0.45-2.31	.96	…	…	…
ARDS (COVID-19)	3.19	1.36-7.50	<.01	0.18	0.03-1.20	.08
COPD	0.28	0.06-1.25	.10	…	…	…
PAH	0.49	0.11-2.21	.36	…	…	…
Others	0.73	0.16-3.34	.68	…	…	…
Donor factors						
Age	1.01	0.97-1.04	.73	…	…	…
Female	1.63	0.73-3.68	.24		…	…
Cause of death						
Anoxia	1.00	0.44-2.26	1.00	…	…	…
Head trauma	0.86	0.37-1.96	.72	…	…	…
Others	1.23	0.49-3.10	.66	…	…	…
Intraoperative factors						
Operative time	1.58	1.23-2.02	<.001	1.14	0.81-1.61	.45
Intraoperative blood transfusion; pRBC	1.18	1.10-1.27	<.001	1.06	0.85-1.33	.61
Intraoperative blood transfusion; FFP	1.26	1.12-1.42	<.001	0.96	0.73-1.25	.75
Intraoperative blood transfusion, PLT	1.71	1.32-2.21	<.001	1.17	0.80-1.71	.42
Ischemic time	1.08	0.86-1.36	.49	…	…	…
VA ECMO use	2.85	1.03-7.91	.04	…	…	…
VA ECMO time	1.02	0.72-1.46	.91	…	…	…

ARDS, acute respiratory distress syndrome; BMI, body mass index; BSA, body surface area; COPD, chronic obstructive pulmonary disease; COVID-19, coronavirus disease 2019; ECMO, extracorporeal membrane oxygenation; FFP, fresh frozen plasma; ILD, interstitial lung disease; LAS, lung allocation score; OR, odds ratio; PAH, pulmonary artery hypertension; VA, venoarterial.

## Comment

The results of this retrospective study provide insights into the association between preoperative blood transfusions and PGD in lung transplant recipients. We observed a significant relationship between preoperative blood transfusions and an increased risk of PGD, as demonstrated by the Cox regression analyses. One of the findings of this study is the identification of preoperative blood transfusions as a risk factor for PGD after lung transplantation. The collective evidence suggests that preoperative blood transfusions play a role in the development of PGD and should be considered during pretransplantation evaluation and management strategies.^3^^,^^5^ The observed association between preoperative blood transfusions and an increased risk of PGD may be attributed to several factors. Transfused blood components can lead to immunologic reactions, including cytokines and donor-specific human leukocyte antigen (HLA) antibodies, endothelial dysfunction, and inflammation, all of which have been implicated in the pathogenesis of PGD.^7^ In this study, blood transfusions within 4 weeks increased the risk of PGD. Additionally, transfusion-related factors such as timing, volume, characteristics of the blood products, and coagulopathy may influence the development of PGD. Ischemic times are also reported to be risk factors for PGD.^8^ Our data demonstrated similar results.

Several limitations should be acknowledged. First, the retrospective and single-institutional design introduces inherent limitations, such as potential selection bias and incomplete data. Second, we can suggest only a limited causal effect of specific transfusion-related factors given the data limitations. This may have been influenced by the number of lung transplants resulting from COVID-19–related acute respiratory distress syndrome, with preoperative ECMO use in patients with pretransplantation blood transfusions.^8^, ^9^, ^10^ Finally, because of the limited number of patients with COVID-19, COVID-19 was not an independent predictor of PGD grade 3 development.

In conclusion, this retrospective study provides evidence supporting the association between preoperative blood transfusions and an increased risk of PGD after lung transplantation. Clinicians should carefully evaluate the necessity of transfusions and explore alternative strategies to minimize the risk of PGD. Further research is warranted to elucidate the underlying mechanisms and guide evidence-based practices in lung transplantation.

## Figures and Tables

**Supplemental Figure:**