# Supplementary material for: The Risk of Pretransplant Blood Transfusion for Primary Graft Dysfunction After Lung Transplant
Source: Ann Thorac Surg Short Rep. 2024 Mar 5;2(3):573–7. doi: 10.1016/j.atssr.2024.02.004 (PMC11708632; doi:10.1016/j.atssr.2024.02.004)
Supplement: Supplemental Material [file mmc1.docx]

*Definition of Complication*

*PGD*

Patients with no evidence of pulmonary edema on chest X-ray (CXR) were considered grade 0. The absence of invasive, mechanical ventilation was graded per the PaO2/FiO2 ratio, using methods similar to those used for mechanical ventilation. If PaO2 was not available for calculation of the PaO2/FiO2 ratio, then an oxygen saturation/FiO2 ratio was used. Grade 1: PaO2/FiO2 ratio >300; Grade 2: PaO2/FiO2 ratio is 200-300; Grade 3: PaO2/FiO2 ratio <200. The lowest PaO2/FiO2 ratio within 72 hours after lung transplantation was used. The use of ECMO for bilateral pulmonary edema on CXR images was classified as grade 3. Continuous use of ECMO without pulmonary edema on CXR imaging was excluded.

*AKI*

AKI was defined using the Risk, Failure, Loss of kidney function, and End-stage kidney disease classification based on prior publications.

*ECMO Indication Criteria*

Prior to lung transplantation, all intubated patients were treated by a multidisciplinary team in accordance with the guidelines of the National Heart, Lung, and Blood Institute’s ARDS Network. Indications for ECMO evaluation included refractory hypoxemia with PaO_2_ less than 55 mmHg, pulse oximetry oxygen saturation less than 88%, and a pH level less than 7.2. This was despite lung-protective, mechanical ventilation with a plateau pressure of less than 35 mmHg, neuromuscular blockade, and prone positioning, in accordance with recommendations from the Extracorporeal Life Support Organization. The decision to initiate ECMO was made by a multidisciplinary team of pulmonologists, thoracic surgeons, ECMO specialists, and intensivists using teleconferencing lines. All procedures were performed by experienced thoracic surgeons in a hybrid operating room under transesophageal echocardiographic and fluoroscopic guidance. The cannula was connected to either a Cardiohelp^TM^ (Getinge, Gothenburg, SE) or CentriMag^TM^ (Abbott, Abbott Park, IL) ECMO circuit.

**SUPPLEMENTAL FIGURES LEGENDS**

**Supplemental Figure.** Identification of study cohort.
